# The Molecular Biology of Cancer

**DOI:** 10.1038/sj.bjc.6603379

**Published:** 2006-10-17

**Authors:** V Emuss

**Affiliations:** 1 Institute of Cancer Research, London, UK

The accumulation of knowledge associated with cancer biology continues to progress at a phenomenal rate; the electronic availability of research papers and news items makes access to new discoveries almost instantaneous. International meetings present opportunities to share scientific knowledge and develop collaborations, as well as fuelling competition between groups, to drive research forward. As a primary researcher or graduate student, the ability to adapt research projects to take advantage of new technological and scientific developments in the field of cancer biology requires a broad knowledge of subjects outside the primary areas of research. Similarly, an undergraduate or newcomer to the field must initially familiarise themselves with established principles before facing the daunting task of processing the vast amount of specialised knowledge available. The editors of *The Molecular Biology of Cancer* have identified the need for a book that assimilates critical aspects of cancer biology into a single resource to function as a reference tool and teaching aid applicable to both students and researchers alike.

The book is divided into 18 chapters and a glossary. Each chapter is authored by experts in the field and is prefaced by a list of the key points covered in that section. The chapters are organised according to a common format; of particular note is the arrangement of the bibliographies at the end of each chapter where references are grouped according to the concepts discussed. This format facilitates navigation of the information presented in each section, allowing the book to be used as a reference tool. This is ideal for established cancer biologists requiring clarity of specific subjects as well as those using the book to learn topics from first principles. It is in catering to this dual audience that the book is particularly strong.

The Introduction presents cancer as a genetic disease that requires the acquisition of the classical ‘hallmarks’ by cancer cells to promote tumour development. Basic concepts are clearly presented in this chapter that would be redundant to an established cancer biologist but invaluable to a newcomer to the field and of great importance in the context of the book as a teaching resource. Chapter 2 considers the clinical features of lung, breast and prostate cancer and determines the context in which the molecular biology described in the subsequent chapters should be considered; namely, in terms of disease manifestation.

The interplay between the environment and genetic lesions associated with cancer is considered in Chapter 3; the book then focuses on the molecular basis of the cellular processes relevant to the cancer cell. The mechanisms fundamental to the normal cell cycle and DNA replication are described in Chapter 4; Chapters 6, 7 and 10 discuss the role of oncogenes, tumour suppressor genes and genetic instability, respectively. Chapter 11 describes the role of epigenetic factors in gene expression and the influence of post-translational modifications on protein expression. There is, however, comparatively little discussion of the role of signal transduction in cancer development. The vast nature of this field makes consideration of every pathway impossible in this context but a more thorough overview could be achieved if a whole chapter were devoted to this topic. The RAS – MAPK pathway and PI3K – Akt pathway are highlighted as examples of critical signalling modules in cell growth as part of Chapter 5 but could be included on their own. This is especially relevant given the potential of signalling pathway components as drug targets.

At times, the complex nature of the mechanisms and theories discussed necessitates detailed descriptions resulting in large blocks of text. However, large, monochrome figures clearly summarise these descriptions or are used in place of them where possible. Additional colour plates illustrate signalling pathways and clinical images that would be unclear in black and white. Information that is relevant to the chapter but would be incongruous in the main text, such as highlights of key experiments, is presented in additional text boxes. Tables are also used to present lists of information, such as protein classifications, in an organised format that would not be possible in the main text. The conclusion of each chapter summarises the topics covered and suggests questions that still remain in the field.

Chapters 15, 16 and 17 address the issues of cancer diagnosis, treatment and patient care and set the book apart from a general molecular biology reference by considering the clinical implications of understanding this subject. Chapter 17 is especially poignant in its highly informative discussion of the management of the palliative care of cancer patients. The title of the book does not imply that these topics would be included; however, it is information I feel is essential to complete the comprehensive reference guide that the editors aimed to produce. The development of therapies specifically targeting the molecules responsible for the transformed phenotype demands a detailed understanding of the mechanisms behind normal cellular processes. This knowledge should not be gained at the expense of an appreciation of the impact that bench-based research has on the patient in hospital. An awareness of the human implications of gaps in our understanding of cancer development should drive research.

The pace of cancer research means that the latest knowledge of highly specialised concepts is essential but, equally, the cross talk between different subject areas means a solid understanding is essential to take advantage of the plethora of new data available. Essentially, *The Molecular Biology of Cancer* is a book that can be used as an introduction to the subject as well as a platform on which to assimilate new information in the context of established principles. This book is applicable to both graduate and undergraduate students and, in the context of a research laboratory, this book would be an excellent resource as a reference guide for scientists at all levels.

